# Evolution of shared care networks by race and ethnicity: findings from the National Health and Aging Trends Study

**DOI:** 10.1093/geronb/gbaf147

**Published:** 2025-08-07

**Authors:** Alexa Bragg, Theodore J Iwashyna, Chanee D Fabius

**Affiliations:** Department of Health Policy and Management, Johns Hopkins Bloomberg School of Public Health, Baltimore, Maryland, United States; Department of Health Policy and Management, Johns Hopkins Bloomberg School of Public Health, Baltimore, Maryland, United States; Department of Medicine, Johns Hopkins School of Medicine, Baltimore, Maryland, United States; Department of Health Policy and Management, Johns Hopkins Bloomberg School of Public Health, Baltimore, Maryland, United States

**Keywords:** Caregiver networks, Task-sharing, Community-based care

## Abstract

**Objectives:**

The aging population in the United States is increasingly diverse, particularly among community-dwelling individuals with disabilities. Black and Hispanic older adults experience greater reliance on assistance with daily activities (e.g., bathing, shopping, banking) than White adults. Assistance is often provided by multiple caregivers helping with the same tasks (“role-sharing”). We describe patterns of shared care by examining racial and ethnic differences in role-sharing in 2015 and 2022.

**Methods:**

We use weighted, repeated cross-sectional data from the 2015 and 2022 National Health and Aging Trends Study, focusing on Black, White, and Hispanic community-dwelling care recipients (*n* = 3,050 in 2015; *n* = 2,318 in 2022) who receive assistance with household (e.g., shopping), self-care (e.g., bathing, dressing), mobility (e.g., getting around indoors), or medical activities (e.g., medication management) due to health or functioning limitations. Logistic regression models using generalized estimating equations assessed race differences in experiencing role-sharing, adjusting for older adult characteristics and survey year.

**Results:**

Role-sharing was most common in the household assistance domain, with approximately 30% of all groups reporting role-sharing in both years. In fully adjusted models, Hispanic recipients experienced lower odds of role-sharing for household tasks (OR: 0.72, 95% CI: 0.57–0.91) compared to White care recipients. Racial and ethnic differences were not observed in other care domains, and role-sharing prevalence was consistent in 2015 and 2022.

**Discussion:**

Findings underscore the complexity of caregiving networks and inform strategies to improve collaboration and reduce role ambiguity among role-sharing caregivers.

The aging population in the United States (U.S.) is becoming increasingly diverse, particularly among those living with disabilities residing in community settings (Okoro et al., 2018). Projections from the U.S. Census Bureau indicate that between 2022 and 2040, the population of individuals aged 65 and older from racial and ethnic minoritized groups is expected to grow by approximately 83%, with significant increases among Hispanic and Black subpopulations (U.S. Census Bureau, 2023). Understanding care networks within the context of these demographic shifts is important, as Black and Hispanic older adults face disproportionately higher rates of cognitive impairment and chronic disease-related physical disabilities compared to their White counterparts (Bishop et al., 2022; Power et al., 2021). These disparities are further compounded by evidence that older adults from racially and ethnically minoritized groups are disproportionately affected by unmet care needs, particularly in accessing critical long-term services and supports such as personal care, adult day services, and caregiver support programs (Fabius, Parikh, et al., 2024; Lin & Liu, 2024). Recent trends in later-life functioning also coincide with changes in the caregiving landscape, as evolving family structures and care arrangements reflect both continued reliance on unpaid family caregivers and growing use of paid care (Miller et al., 2024; Van Houtven et al., 2020; Wolff et al., 2025).

Care networks evolve as an individual’s functional limitations progress, leading to an increased reliance on family caregivers (Favreault et al., 2023). Although immediate family members often provide intensive support, caregiving arrangements can be dynamic and multifaceted, frequently involving extended family members, friends, community networks, and paid caregivers (Hu et al., 2023). Caregiver network typologies may range from a partnership model, where two caregivers equitably share caregiving tasks (Keith, 1995), to distributed and more extensive networks involving non-family members that provide varying levels of support (Ellis et al., 2023; Ellwardt et al., 2016). Additionally, older adults from racially and ethnically minoritized groups often hold strong cultural motivations for family caregiving motivations (e.g., sense of duty, obligation) and are more likely than White older adults to receive help from a child or other family member (Dilworth-Anderson et al., 2020; Nkimbeng & Parker, 2021; Parker & Fabius, 2022). Importantly, racial and ethnic differences shape the structure and demands of these care networks. For example, Black individuals tend to have more kin affected by dementia, increasing the complexity and intensity of caregiving responsibilities (Feng et al., 2024; Lai et al., 2025). Racial and ethnic disparities also extend into health care-related caregiving tasks. In a nationally representative sample, Black and Hispanic caregivers were more likely than White caregivers to assist with medical care activities such as managing special diets and exercises (Leggett et al., 2022).

A growing body of literature underscores the complexity of caregiving arrangements, such as the dynamic between paid and unpaid supports provided to care recipients (Shaw et al., 2021), the proximity and availability of caregivers (Patterson & Margolis, 2023), the willingness to provide care (Canell & Caskie, 2022), overlapping caregiving roles (i.e., network multiplexity) (Shang & Patterson, 2024). Although there are virtues to simplifying care needs as if they were a unidimensional construct, the reality is more complicated. Caregivers frequently experience “role-sharing,” defined in this study as the involvement of multiple caregivers assisting with the same caregiving tasks for a given care recipient, to collectively ensure the provision of support (Ali, McAvay, et al., 2022; Ellis et al., 2023; Spillman et al., 2021). Although prior studies have documented role-sharing specifically among caregivers of individuals with dementia (Fabius, Gallo, et al., 2024; Marcum et al., 2020; Spillman et al., 2020), a recent study using a nationally representative sample found that shared caregiving is common more broadly; nearly half (49%) of older adults receive help from multiple caregivers, even when a single “designated” primary caregiver is identified (Hu et al., 2023). Given the complexity of caregiving needs and how they are met, this article explores role-sharing practices across domains of household (e.g., laundry, banking, shopping), self-care (e.g., eating, bathing), mobility (e.g., getting around inside), and medical assistance (e.g., help with doctor’s visits) among individuals receiving assistance due to health or functional impairments. Examining role-sharing across these domains offers a holistic view of caregiving, revealing how caregiver networks are shaped by the alignment between the specific tasks or services needed and the availability of caregivers.

## Conceptual framework

This article focuses on the type of caregiving tasks performed and the overall structure of the caregiving network, particularly in terms of how roles are shared. We utilize Litwak’s Task-Specific Model of Helper Selection (Litwak, 1985) as a grounding framework to explore how role-sharing patterns vary across different racial and ethnic groups between two time points. The Litwak Model provides a structure for understanding care collaboration, where different caregivers assume complementary or shared roles, each specifically tailored to address the unique tasks or needs of the care recipients. Key factors influencing caregiver-task fit include proximity, availability, similarity in lifestyle or social roles, and the size of the caregiving network. The model also considers the complex activities essential for independent living—such as banking and meal preparation—providing a more comprehensive perspective that includes not only activities of daily living (ADLs) (e.g., bathing, dressing, eating) as predictors of health and healthcare utilization but also instrumental activities of daily living (IADLs) (e.g., meal preparation, laundry) which are critical for maintaining independence. For instance, close family members might offer emotional support or help with medication management, while neighbors or community members might assist with practical tasks like transportation or running errands. By recognizing that role-sharing practices vary based on the specific tasks and the distinct forms of collaboration they entail, this model enables us to examine role-sharing in a more granular way.

Caregiving arrangements are also dynamic, shaped by personal characteristics (e.g., race and gender) and situational factors (e.g., unpredictable life events, age-related changes in physical or cognitive abilities) (Antonucci & Akiyama, 1987). Building on this foundation, researchers have expanded Litwak’s Model to account for the interplay between gender and care across the life course, demonstrating that caregiving arrangements are dynamic and evolve in response to the changing needs of care recipients as well as the shifting availability, capacity, and willingness of caregivers (Allen et al., 2012). In addition, the composition of care networks may evolve in ways that reflect broader demographic, cultural, and psychosocial trends (Manalel & Antonucci, 2022). These external and situational factors influence social relationships (e.g., presence or absence of role-sharing), which, in turn, affect the provision and receipt of support. The onset of the Coronavirus Disease 2019 (COVID-19) pandemic and the implementation of “social distancing” measures provide a salient example of how such time-varying factors may influence care arrangements. For example, Moon et al. (2022) found that during the pandemic, compared to the pre-pandemic period, White caregivers of older adults with dementia significantly increased their assistance with ADLs, while Black caregivers experienced greater increases in providing IADL assistance (Moon et al., 2022).

These findings further underscore the role of race and ethnicity in shaping caregiving patterns and the structure of care networks. Although White older adults are more likely to receive care from spouses and rely on assistive technologies for self-care, Black and Hispanic care networks often reflect broader, more communal caregiving structures (Lin, 2025; York Cornwell & Goldman, 2021). For example, Black social networks may be smaller compared to their White counterparts, but they are more likely to include extended family, fictive kin (e.g., godparents), friends, and local church members (Ajrouch et al., 2001; Lin, 2025; Taylor et al., 2021). Hispanic older adults are also more likely to report kin-centered (i.e., family-focused) networks and maintain more frequent contact with their social ties (Ali, Elliott, et al., 2022). Accounting for shared care in the study of care arrangements yields meaningful insights, as empirical evidence demonstrates that individuals with diverse social networks—including partners, family members, and friends—exhibit superior health outcomes, such as enhanced functional health (Ali et al., 2018).

To our knowledge, no studies have explicitly explored patterns of role-sharing in caregiving across racial and ethnic groups living with functional limitations. Guided by the task specificity model, we examine racial and ethnic differences in the presence of role-sharing among community-dwelling care recipients who reported receiving assistance with ADLs and IADLs in 2015 and 2022. Drawing on documented differences in caregiving network characteristics across diverse communities, we hypothesize that (a) there are racial and ethnic differences in the presence of shared care within Black, Hispanic, and White care recipients’ networks, and (b) the prevalence of role-sharing in 2015 and 2022 varies by race and ethnicity. Such variation may reflect broader shifts in care needs and the availability of support networks over time. Recognizing the critical role of robust social support networks in addressing the unmet needs of care recipients, this study aims to inform the development of policies and interventions that accommodate diverse caregiving networks and arrangements.

## Method

This study examines two cross-sectional cohorts of community-dwelling older adults from the nationally representative study of Medicare beneficiaries, National Health and Aging Trends (NHATS), who reported receiving assistance with either household, self-care, mobility, or medical tasks. Using data from the 2015 and 2022 survey waves, we examine how role-sharing patterns within caregiving networks vary by race and ethnicity and compare the prevalence of role-sharing across cohorts to assess the consistency of caregiving arrangements over time.

### Data and sample

National Health and Aging Trends is a nationally representative study of Medicare beneficiaries aged 65 and older (Freedman et al., 2019). Initially administered in 2011, the survey provides detailed information regarding the participants’ current living arrangements, demographics, socioeconomic status, health and cognitive functioning, and type of assistance received by the sampled older adult in the past month. A separate Other Person file (linked by a common participant identification variable) compiles a cumulative roster of helpers identified by the older adult and documents their relationship and type and amount of assistance provided to the NHATS participant. Study participants are re-interviewed annually. We selected the 2015 and 2022 waves of NHATS to support a repeated cross-sectional analysis of caregiving networks across two strategically important time points. Both survey rounds followed sample replenishments and oversampled Black older adults and those aged 85 and older. The 2022 wave oversampled Hispanic individuals. Individuals were included in the analytic sample for each year if they reported receiving any help with household (laundry, shopping, meal preparation, banking), self-care (bathing, dressing, eating, toileting), mobility (getting around in and outdoors, transferring in and out of bed), or medical tasks (medication administration, received help with doctor appointments) for health or functioning reasons in the last month, resided in the community, and identified their primary race and ethnicity as non-Hispanic Black, non-Hispanic White, or Hispanic. Of the 8,334 sample persons surveyed in 2015 and the 6,087 sample persons surveyed in 2022, only 3,050 and 2,318 sample persons, respectively, fulfilled these criteria and were retained for subsequent analysis (Supplementary Figure 1). Following NHATS guidance, round-specific analytic weights were applied to account for the complex sampling design and ensure representativeness of each cross-section.

### Measures

#### Sociodemographic characteristics

We included several sociodemographic characteristics of care recipients. Age was categorized as 65–74 (reference), 75–84, or 85 and older, gender (coded 1 for female and 0 for male), income level, and self-reported Medicaid enrollment at time of interview (coded 1 for yes and 0 for no). Total income data from NHATS, reported for the prior calendar year in Rounds 5 and 12, were used to derive respondents’ federal poverty level (FPL) status. To address item nonresponse, NHATS provides multiply imputed income values (using a hot deck method in Round 5 and interval regression in Round 12) (Hu & Freedman, 2024). In accordance with NHATS documentation, imputed outliers were treated as missing, and the first imputed value was substituted for respondents with otherwise missing income data. FPL was calculated by dividing household income by the relevant U.S. Census Bureau poverty threshold for adults aged 65 and older in 2015 and 2022 (U.S. Census Bureau, 2025). Individuals were classified into three poverty categories: below 100% of the FPL (reference), 100%–200%, and above 200%.

#### Health and function characteristics

We include measures reflecting the level of assistance and probable dementia. Level of assistance was categorized into three mutually exclusive levels: assistance with household activities only (reference); assistance with one to two self-care or mobility activities; or assistance with three or more self-care or mobility activities (Martinson & Berridge, 2015). Probable dementia (coded 1 for yes and 0 for no) was ascertained using a validated classification approach developed by Kasper et al. (2013), which incorporates multiple sources of information: a self- or proxy-reported diagnosis of dementia or Alzheimer’s disease, a score of ≥2 on the AD8 Dementia Screening Interview, or cognitive test scores indicating impairment (Kasper et al., 2013).

#### Care circumstances

We include measures reflecting hours of care received per week and living arrangement (living alone [reference], with a spouse/partner only, with a spouse/partner and others, or with others only). The calculation of hours of care followed the imputation algorithm outlined in the NHATS documentation (Freedman et al., 2014). This algorithm provided estimates of monthly caregiving hours, which were subsequently converted to a weekly basis by dividing the total monthly hours by the number of weeks in a month. We created a three-category measure to reflect whether someone was receiving care amounting to 20 or fewer hours (reference), 21–39 hours, or 40 or more hours per week.

#### Helper characteristics

Helper characteristics include relationship to the care recipient, gender, co-residence within the household, and paid versus unpaid status. We categorized relationships to the care recipient into four groups: spouse/partner, child, other family member, and nonrelative. We coded gender as female and male, co-residence as living in the same household as the care recipient versus not, and paid status as paid helper versus unpaid.

#### Exposure: Race and ethnicity

In this study, we treat race and ethnicity as the primary “exposure” of interest, recognizing it as a social construct. We used the NHATS-derived categorical variable for sampled persons’ self-reported race and ethnicity: non-Hispanic White (hereafter “White”), non-Hispanic Black (“Black”), and Hispanic or Latinx.

#### Outcome: Role-sharing

The NHATS includes questions about who provides assistance for those who indicate they received help with specific activities. We categorize caregiving tasks (Litwak, 1986; Spillman et al., 2020) into four domains: household (laundry, banking, shopping, meal preparation, and money management), self-care (eating, bathing, toileting, dressing), mobility (getting around inside, getting around outside), and medical (medication management, doctors’ visits) assistance. In the Other Person file (refer to “Data and Sample” section), each caregiver linked to a care recipient reports the specific tasks they assist with, allowing us to classify the type of assistance provided by domain (e.g., self-care, mobility). Using these data, we categorized care recipients in each survey round based on whether they received help from no caregivers, one caregiver, or two or more caregivers within each care domain. Importantly, classification of “no caregivers” reflects cases in which the care recipient did not receive hands-on assistance in that specific domain.

### Statistical analysis

This study uses a repeated cross-sectional design, drawing on two independent survey waves (2015 and 2022) of the NHATS. Although some individuals may appear in both rounds due to NHATS’s longitudinal structure, our analyses do not track individual-level change over time. Instead, we examine whether patterns of role-sharing differ by race and ethnicity and assess whether these patterns remain consistent across the two time points. To examine racial and ethnic differences in the characteristics of older adults and their caregivers, we first compared the weighted estimates of demographic and socioeconomic variables across racial and ethnic groups using Pearson chi-square tests for categorical variables. We also used chi-square tests to assess group differences in the number of helpers within each care domain, categorized as no helpers, one helper, or two or more helpers. These descriptive analyses were conducted cross-sectionally for 2015 and 2022 and provided estimates of caregiving network size, as well as the proportion of care recipients receiving assistance from multiple individuals within each domain. To assess differences in role-sharing across racial and ethnic groups, we used generalized linear models fit with generalized estimating equations (GEEs), treating the outcome, role-sharing, as a binary indicator (two or more vs. one caregiver) for each care domain. These models were estimated using a pooled dataset combining the 2015 and 2022 survey waves. These domain-specific models only included respondents who reported receiving help in the relevant domain. For regression models, we also generated a continuous variable, “number of assisted activities,” representing the total number of reported activities for which the respondent received help (range: 0–13). This count was included in generalized estimating equations models to account for variation in overall care intensity. Although generalized estimating equation accounts for clustering of repeated observations among individuals appearing in both years, we estimate population-average associations in a cross-sectional framework and do not infer within-person change. To preserve the nationally representative structure of the data, we applied NHATS-provided analytic weights appropriate for each wave and incorporated survey design variables (strata and primary sampling units) into all models.

All models were adjusted for care recipients’ sociodemographic factors (income, age, gender, Medicaid coverage), health characteristics (dementia, amount of assistance), and care circumstances (hours of care, living arrangement). Separate sensitivity analyses were conducted, omitting care recipients who received paid help to assess the robustness of the findings. All analyses were conducted in STATA 18.0 using survey sampling weights. Statistical significance determined by a two-sided *p*-value of .05.

## Results

### Descriptive results

Although few characteristics of care recipients changed between 2015 and 2022, a greater proportion of Black and Hispanic older adults were female, lived below the federal poverty line, were enrolled in Medicaid, and experienced dementia (Table 1). Among Hispanic older adults, the total hours of care received were notably high, with 35.4% in 2022 and 31.4% in 2015 reporting 40 or more hours of care per week. In 2022, a greater proportion of Black older adults reported living alone (30.6%) compared to 2015 (24.0%), while a smaller proportion reported cohabiting with others only (33.0%) compared to 2015 (37.5%). Helper characteristics for older adults in the analytic sample are detailed in Supplementary Table 1, with a notable decline in the proportion of children identified as helpers between 2015 and 2022, except among Hispanic care recipients, where this proportion remained stable. Across both years, approximately half of care recipients received care from two or more helpers.

**Table 1.** Sociodemographic, health, and care circumstance characteristics of care recipients in 2015 and 2022.

Variable	2015				2022			
	White	Black	Hispanic	*p*-value	White	Black	Hispanic	*p*-value
**Weighted estimates (*thousands*)**	11,904.10	1,396.52	1,495.28	.00	12,504.96	1,781.39	1,859.62	.00
**Unweighted, *n***	2,039	755	256		1,377	580	361	
**Sociodemographic, % (*n*)**								
** Age (years)**				.63				.00
** 65–74**	44.3 (538)	45.6 (214)	49.9 (78)		46.8 (291)	52.2 (142)	48.5 (108)	
** 75–85**	37.1 (869)	36.1 (312)	33.7 (103)		37.1 (600)	32.4 (268)	36.0 (160)	
** 85+**	18.6 (632)	18.3 (229)	16.4 (75)		16.1 (486)	15.3 (170)	15.5 (93)	
** Gender**								.00
** Female**	52.2 (1,122)	62.7 (496)	56.8 (151)	.00	52.2 (766)	62.1 (381)	60.1 (231)	
** Federal poverty level**								.00
** <100%**	10.9 (245)	35.4 (286)	48.9 (142)	.00	12.6 (155)	43.2 (244)	55.5 (206)	.00
** 100%–200%**	24.2 (550)	32.0 (257)	33.5 (79)		23.8 (363)	26.7 (177)	25.4 (103)	
** 200%+**	64.9 (1,244)	32.5 (212)	17.7 (35)		63.7 (859)	30.1 (159)	19.2 (52)	
** Medicaid eligible**	9.3 (168)	39.9 (291)	53.5 (133)	.00	10.8 (121)	41.2 (231)	56.5 (201)	
Health and function, % (*n*)								.00
Level of assistance				.00				.00
Household only	62.7 (1223)	52.0 (365)	53.1 (124)		59.9 (789)	47.7 (257)	42.9 (155)	
1–2 Self-care/mobility	24.3 (516)	28.8 (219)	24.3 (65)		26.6 (387)	32.6 (186)	31.1 (111)	
3+ Self-care/mobility	13.0 (200)	19.2 (171)	22.7 (67)		13.6 (201)	19.8 (137)	25.9 (95)	
Probable dementia	13.0 (335)	21.0 (206)	25.1 (82)	.00	11.2 (188)	18.1 (144)	21.2 (99)	
Care circumstance, % (*n*)								
Hours of care per week								.00
20 hours or fewer	65.8 (1277)	55.9 (393)	47.5 (110)	.00	72.5 (945)	56.8 (319)	44.9 (163)	
21–39 hours	17.1 (358)	17.8 (128)	21.1 (54)		12.9 (200)	17.7 (101)	19.7 (70)	
40 hours or more	17.1 (404)	26.4 (234)	31.4 (92)		14.6 (232)	25.5 (160)	35.4 (128)	
Living arrangement								.00
Alone	20.4 (519)	24.0 (197)	15.0 (43)	.00	20.3 (341)	30.6 (166)	15.8 (57)	
Spouse/partner only	52.7 (954)	26.2 (156)	33.1 (68)		52.6 (659)	24.1 (113)	25.0 (87)	
Spouse/partner and others	12.0 (211)	12.6 (84)	19.5 (44)		11.9 (120)	12.2 (71)	20.7 (69)	
With others only	14.9 (355)	37.2 (318)	32.4 (101)		15.3 (257)	33.0 (230)	38.6 (148)	

**Figure 1.**
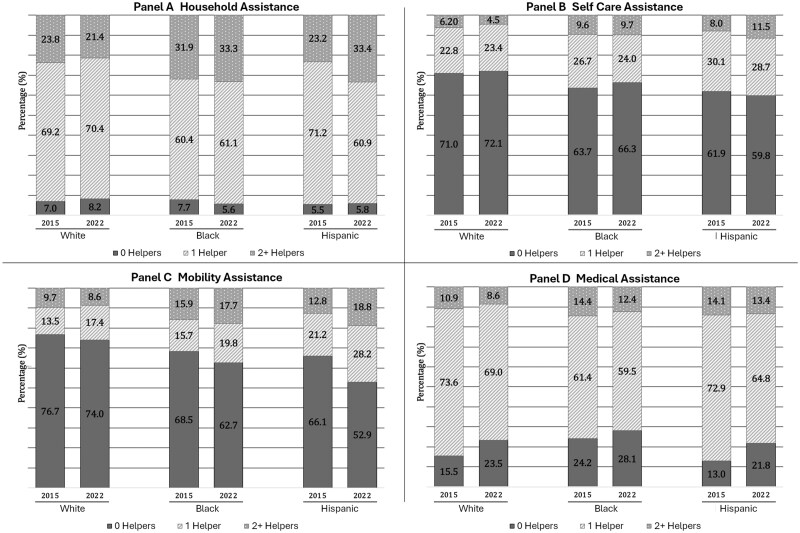
Proportion of older adults who have 0, 1, or 2+ caregivers by domain in 2015 and 2022.

The greatest amount of role-sharing was observed in household tasks; roughly 30% of Black, White, and Hispanic older adults reported assistance from two or more caregivers in both 2015 and 2022 (Figure 1). In contrast, the majority of care recipients depended on a single helper for medical-related assistance; however, the proportion of Black care recipients who did not receive any medical-related assistance (24.2% in 2015 and 28.1% in 2022) was higher than that of their White and Hispanic counterparts. Chi-square tests also revealed statistically significant relationships between race and ethnicity and care arrangements across all care domains and time points (Supplementary Table 2). Black and Hispanic care recipients were more likely to experience role-sharing for self-care, mobility assistance, and medical assistance, compared to White care recipients.

### Associations between race and ethnicity and role-sharing in 2015 and 2022

Among older adults who received assistance with household, self-care, mobility, or medical assistance, racial and ethnic differences were identified in univariate regression analyses (Table 2). Black care recipients had higher odds of experiencing role-sharing for household (odds ratio [OR]: 1.62, 95% confidence interval [CI]: 1.41–1.87), self-care (OR: 1.44, 95% CI: 1.13–1.82), mobility (OR: 1.44, 95% CI: 1.16–1.79) and medical assistance (OR: 1.51, 95% CI: 1.26–1.81) compared to White care recipients. Similarly, Hispanic care recipients also exhibited higher odds of experiencing role-sharing for household (OR: 1.24, 95% CI: 1.03–1.51), self-care (OR: 1.41, 95% CI: 1.03–1.93), and medical assistance (OR: 1.37, 95% CI: 1.07–1.76) compared to White care recipients. These differences were attenuated in adjusted models for medical, self-care, and mobility assistance. Among those receiving help with household activities, Hispanic care recipients had significantly lower odds of experiencing role-sharing compared to White care recipients (OR: 0.72, 95% CI: 0.57–0.91). We did not observe significant differences in role-sharing by survey year. Sensitivity analyses corroborated these findings, showing consistent results both when care recipients who received paid assistance were excluded and when total hours of care and gender were omitted from the model.

**Table 2.** Generalized estimating equation logistic regression of race and ethnicity predicting the presence of role-sharing using pooled 2015 and 2022 NHATS data.

Variable	Household assistance		Self-care assistance		Mobility assistance			Medical assistance		
	(OR (95% CI))		(OR (95% CI))		(OR (95% CI))			(OR (95% CI))		
Care recipient race: White (ref)										
Black	1.62*** (1.41, 1.87)	1.02 (0.86, 1.22)	1.44** (1.13, 1.82)	1.00 (0.73, 1.37)		1.44** (1.16, 1.79)	1.22 (0.93, 1.59)		1.51*** (1.26, 1.81)	1.09 (0.87, 1.37)
Hispanic	1.24* (1.03, 1.51)	0.72*** (0.57, 0.91)	1.41* (1.03, 1.93)	0.81 (0.53, 1.23)		1.00 (0.76, 11.32)	0.93 (0.65, 1.32)		1.37** (1.07, 1.76)	1.03 (0.76, 1.39)
Sociodemographic characteristics										
Care recipient age: 65–74 years (ref)										
75–84 years		1.23** (1.03, 1.46)		1.24 (0.86, 1.78)			1.19 (0.88, 1.60)			1.40** (1.08, 1.82)
85+ years		1.45*** (1.19, 1.76)		1.36 (0.93, 1.99)			1.32* (0.96, 1.83)			2.08*** (1.58, 2.73)
Care recipient gender (female)		1.43*** (1.23, 1.67)		1.24 (0.93, 1.65)						1.51*** (1.23, 1.86)
Probable dementia (yes)		1.17 (0.93, 1.49)		0.85 (0.57, 1.25)			1.21 (0.86, 1.72)			1.13 (0.84, 1.50)
Medicaid (yes)		0.99 (0.82, 1.20)		1.08 (0.79–1.49)			0.75** (0.57, 1.00)			1.02 (0.79, 1.30)
Federal poverty level (FPL): <100% FPL (ref)										
100%–200% FPL		1.16 (0.95, 1.41)		1.20 (0.85, 1.69)			0.92 (0.69, 1.24)			1.13 (0.88, 1.47)
200% FPL+		1.09 (0.88, 1.36)		1.20 (0.82, 1.78)			1.04 (0.74, 1.45)			1.04 (0.78, 1.38)
Number of assisted activities		1.14*** (1.12, 1.17)		1.33*** (1.27, 1.39)			1.20*** (1.15, 1.25)			1.14*** (1.11, 1.18)
Hours of care: 20 hours or fewer (ref)										
21–39 hours		1.25** (1.04, 1.51)		0.98 (0.65, 1.48)			0.77 (0.56, 1.05)			1.06 (0.81, 1.39)
40 hours or more		1.44*** (1.19, 1.75)		1.45** (1.00, 2.09)			1.33* (0.98, 1.82)			1.57*** (1.21, 2.02)
Living arrangement: Alone (ref)										
Spouse/partner-only		0.35*** (0.29, 0.44)		0.69* (0.46, 1.05)			0.39*** (0.27, 0.55)			0.50*** (0.38, 0.65)
Spouse/partner and others		1.56*** (1.23, 1.97)		1.22 (0.75, 1.98)			1.12 (0.75, 1.67)			0.81 (0.58, 1.12)
With others only		1.04 (0.86, 1.25)		1.05 (0.73, 1.50)			0.86 (0.64, 1.14)			0.67** (0.53–0.85)
Time		1.08 (0.94, 1.24)		1.04 (0.81, 1.33)			0.97 (0.79, 1.20)			0.87 (0.73, 1.04)

*Note*. FPL = Federal Poverty Level; NHATS = National Health and Aging Trends; ref = reference.

*** *p* < .01;

** *p* < .05;

* *p* < .1.

## Discussion

This study contributes to the growing body of literature that moves beyond the traditional focus on a single primary caregiver, underscoring the prevalence of shared caregiving roles across the care network for certain support tasks (Ellis et al., 2023; Hu et al., 2023; Shang & Patterson, 2024). Role-sharing may be an indication of care coordination, which is widely acknowledged as a significant determinant of the health and well-being of older adults (Ayalon & Levkovich, 2019) and may help distribute caregiving responsibilities, thereby alleviating individual burden (Liang et al., 2022). These patterns are consistent with Litwak’s Task-Specific Model of Helper Selection (1985), which posits that caregiving roles are shaped by the nature of the task itself, such as its technical demands, required frequency, and degree of intimacy. As such, certain domains (e.g., household activities) may be more amenable to shared caregiving arrangements than others. We find that, after adjusting for sociodemographic, health, and function, and care circumstances, Hispanic older adults were less likely to experience role-sharing in household activities. Additionally, although recent demographic and family changes have reshaped the caregiving landscape, the prevalence of role-sharing remained stable across the two time points. This lack of temporal variation may be due to the relatively short interval between survey waves or the influence of unmeasured factors not captured in this analysis.

Notably, our findings align with recent research documenting a decline in the involvement of children as identified caregivers, which may contribute to shifts in how care is distributed across networks (Verdery & Margolis, 2017). Our findings have implications for policymakers in designing and implementing policies that promote role-sharing within diverse care networks, such as expanded access to caregiver training, recognition of multiple caregivers in electronic health records, and loosening eligibility requirements for accessing supportive services (e.g., respite).

Our finding that multiple caregivers collaborate to meet the care needs of older adults aligns with both theoretical frameworks (Antonucci & Akiyama, 1987) and empirical evidence (Marcum et al., 2020; Spillman et al., 2020). However, caregiving responsibilities may not be shared uniformly across different types of tasks and may be influenced by factors such as the complexity and time demands of the tasks, cultural perceptions surrounding certain activities, and the availability of caregivers with the appropriate skills to provide assistance. We posit that Hispanic older adults may be less likely to participate in caregiver role-sharing compared to Black and White older adults, potentially due to cultural norms or structural factors that influence caregiving practices within Hispanic care networks. For example, research on caregiving among women of Mexican descent identified a pattern of self-sacrificing behaviors aligned with the cultural ideal of “marianismo,” often resulting in caregiving responsibilities being concentrated on a single individual (mostly women) within the care network (Mendez-Luck & Anthony, 2016). Additionally, it is important to consider the role of acculturation, which may shift caregiving expectations and practices over time, resulting in within-group heterogeneity in Hispanic care networks (Jolicoeur & Madden, 2002). Although Hispanic care recipients are often treated as a homogeneous group in existing research, our findings underscore the need for more nuanced and culturally contextualized approaches to understanding racial and ethnic differences in this population.

We identified role-sharing with household tasks as a modifiable factor essential for promoting the well-being and independence of older adults. Unlike more extensively studied aspects of ADLs, such as toileting or mobility assistance, household tasks often reflect a broader scope of support, including meal preparation, cleaning, and managing finances that are essential for developing proactive care interventions to enable older adults to maintain autonomy and quality of life (Mlinac & Feng, 2016). In addition, our findings suggest that tasks involving complex decision-making (e.g., assistance with medical appointments) or self-management activities (e.g., bathing, toileting) frequently rely on a sole helper, which merits further investigation, particularly with regard to its implications for unmet care needs and heightened caregiver burden (Song et al., 2023). Differences in the distribution of shared care present important implications for long-term care policies, such as the Recognize, Assist, Include, Support, and Engage Family Caregivers Act, as the declining caregiver-to-older-adult ratio and increasing reliance on paid care services necessitate a nuanced understanding of where and how collaboration occurs in the care network (Freedman et al., 2024).

This study has several limitations. First, although we assess role-sharing in caregiving, we are unable to account for the distribution of caregiving responsibilities among multiple caregivers, which may result in an oversimplified portrayal of caregiving collaboration patterns. Second, although NHATS provides comprehensive data on the various sources of care received, it does not capture all potential characteristics of caregivers within each care network, such as their socioeconomic status, cultural background, caregiving experience, emotional well-being, or time availability. Future research is needed to better understand the mechanisms through which different care network characteristics influence the availability and preferences for shared caregiving arrangements (Liang et al., 2022). Third, the conceptualization and measurement of race in this study warrant further reflection and more in-depth examination. The effects of race are likely intertwined with a range of other sociodemographic factors, including socioeconomic status, cultural norms, marital status, and gender, all of which shape caregiving practices and experiences. Finally, although we used weighted repeated cross-sectional data from 2015 and 2022, our analyses reflect population-level patterns at each time point rather than longitudinal trajectories. The precision of our estimates is also constrained by the fact that only the 2022 dataset oversamples Hispanic populations. Despite these limitations, examining role-sharing across various domains of assistance can illuminate where collaboration is most fertile and where it may be more limited.

## Conclusion

Although public and private aging efforts often focus on the individual rather than the household or community, policies that address the collective needs and capacities of care networks are needed. Our findings suggest that although role-sharing is relatively common, particularly among those performing household-related tasks, racial and ethnic differences are evident. These results demonstrate the need for future research to understand the quality of shared care arrangements and their potential implications for caregiver outcomes, as well as culturally tailored interventions to strengthen the diverse caregiving arrangements across different populations.

## Supplementary Material

gbaf147_Supplementary_Data

## Data Availability

The data used in this study are publicly available from the NHATS Study and can be accessed upon registration at https://www.nhats.org/user/register. The analytic methods and code are available from the corresponding author upon request.

## References

[gbaf147-B1] Ajrouch K. J. , Antonucci T. C., Janevic M. R. (2001). Social networks among Blacks and Whites: the interaction between race and age. The Journals of Gerontology, Series B: Psychological Sciences and Social Sciences, 56, S112–S118. 10.1093/geronb/56.2.S112 11245365

[gbaf147-B2] Ali T. , Elliott M. R., Antonucci T. C., Needham B. L., Zelner J., Mendes de Leon C. F. (2022). Multidimensional social network types and their correlates in older Americans. Innovation in Aging, 6, igab053. 10.1093/geroni/igab053 35036584 PMC8756185

[gbaf147-B3] Ali T. , McAvay G. J., Monin J. K., Gill T. M. (2022). Patterns of caregiving among older adults with and without dementia: A latent class analysis. The Journals of Gerontology, Series B: Psychological Sciences and Social Sciences, 77(Suppl_1), S74–S85. 10.1093/geronb/gbab237 35032392 PMC9122635

[gbaf147-B4] Ali T. , Nilsson C. J., Weuve J., Rajan K. B., Mendes de Leon C. F. (2018). Effects of social network diversity on mortality, cognition and physical function in the elderly: A longitudinal analysis of the Chicago Health and Aging Project (CHAP). Journal of Epidemiology and Community Health, 72, 990–996. 10.1136/jech-2017-210236 29970598

[gbaf147-B5] Allen S. M. , Lima J. C., Goldscheider F. K., Roy J. (2012). Primary caregiver characteristics and transitions in community-based care. The Journals of Gerontology. Series B: Psychological Sciences and Social Sciences, 67, 362–371. 10.1093/geronb/gbs032 22492069 PMC3325088

[gbaf147-B6] Antonucci T. C. , Akiyama H. (1987). Social networks in adult life and a preliminary examination of the convoy model. Journal of Gerontology, 42, 519–527. 10.1093/geronj/42.5.519 3624811

[gbaf147-B7] Ayalon L. , Levkovich I. (2019). A systematic review of research on social networks of older adults. The Gerontologist, 59, e164–e176. 10.1093/geront/gnx218 29385450

[gbaf147-B8] Bishop N. J. , Haas S. A., Quiñones A. R. (2022). Cohort trends in the burden of multiple chronic conditions among aging U.S. adults. The Journals of Gerontology, Series B: Psychological Sciences and Social Sciences, 77, 1867–1879. 10.1093/geronb/gbac070 35642746 PMC9535783

[gbaf147-B9] Canell A. E. , Caskie G. I. L. (2022). Emerging adult caregivers: quality of contact, ageism, and future caregiving. The Gerontologist, 62, 984–993. 10.1093/geront/gnab173 34971387

[gbaf147-B10] Dilworth-Anderson P. , Moon H., Aranda M. P. (2020). Dementia caregiving research: expanding and reframing the lens of diversity, inclusivity, and intersectionality. The Gerontologist, 60, 797–805. 10.1093/geront/gnaa050 32667672 PMC7362616

[gbaf147-B11] Ellis K. R. , Koumoutzis A., Lewis J. P., Lin Z., Zhou Y., Chopik W. J., Gonzalez R. (2023). Conceptualizing and operationalizing collaboration among multiple caregivers of older adults. The Journals of Gerontology. Series B: Psychological Sciences and Social Sciences, 78(Suppl 1), S27–S37. 10.1093/geronb/gbac139 36409283 PMC10010467

[gbaf147-B12] Ellwardt L. , Aartsen M., van Tilburg T. (2016). Types of Non-kin networks and their association with survival in late adulthood: a latent class approach. The Journals of Gerontology, Series B: Psychological Sciences and Social Sciences, 72, 694–705. 10.1093/geronb/gbw142 28329792

[gbaf147-B13] Fabius C. D. , Gallo J. J., Burgdorf J., Samus Q. M., Skehan M., Stockwell I., Wolff J. L. (2024). Family care partners and paid caregivers: national estimates of role-sharing in home care. The Gerontologist, 65, gnae177. 10.1093/geront/gnae177 PMC11772859 39657690

[gbaf147-B14] Fabius C. D. , Parikh R., Wolf J. M., Giordano S., Fashaw-Walters S., Jutkowitz E., Shippee T. (2024). Racial and ethnic differences in unmet needs among older adults receiving publicly-funded home and community-based services. Journal of the American Geriatrics Society, 72, 3520–3529. 10.1111/jgs.19153 39210674 PMC11560522

[gbaf147-B15] Favreault M. , Dey J., Anderson L., Lamont H., Marton W. (2023). *Changes in caregiving networks over the course of disability: How family caregiving networks evolve after onset of needs for long-term services and supports (issue brief)*. Office of the Assistant Secretary for Planning and Evaluation (ASPE). 39250583

[gbaf147-B16] Feng K. , Song X., Caswell H. (2024). Kinship and care: racial disparities in potential dementia caregiving in the United States from 2000 to 2060. The Journals of Gerontology. Series A, Biological Sciences and Medical Sciences, 79(Suppl_1), S32–S41. 10.1093/gerona/glae106 38642100 PMC11542221

[gbaf147-B17] Freedman V. A. , Agree E. M., Seltzer J. A., Birditt K. S., Fingerman K. L., Friedman E. M., Lin I.-F., Margolis R., Park S. S., Patterson S. E., Polenick C. A., Reczek R., Reyes A. M., Truskinovsky Y., Wiemers E. E., Wu H., Wolf D. A., Wolff J. L., Zarit S. H. (2024). The changing demography of late-life family caregiving: a research agenda to understand future care networks for an aging U.S. population. The Gerontologist, 64, gnad036. 10.1093/geront/gnad036 36999951 PMC10825830

[gbaf147-B18] Freedman V. A. , Skehan M. E., Hu M., Wolff J. (2019). *National study of caregiving I-III user guide*. Johns Hopkins Bloomberg School of Public Health.

[gbaf147-B19] Freedman V. A. , Spillman B. C., Kasper J. (2014). *Hours of care in rounds 1 and 2 of the national health and aging trends study.* NHATS Technical Paper #7. Johns Hopkins Bloomberg School of Public Health.

[gbaf147-B20] Hu M. , Freedman V. (2024). *National health and aging trends study twenty interval regression income imputations: Rounds 1-13.* NHATS Technical Paper #49. Johns Hopkins Bloomberg School of Public Health.

[gbaf147-B21] Hu M. , Freedman V. A., Patterson S. E., Lewis N. (2023). Shared care networks assisting older adults: new insights from the national health and aging trends study. The Gerontologist, 63, 840–850. 10.1093/geront/gnac155 36190818 PMC10268586

[gbaf147-B22] Jolicoeur P. M. , Madden T. (2002). The good daughters. Journal of Aging Studies, 16, 107–120. 10.1016/S0890-4065(02)00038-5

[gbaf147-B23] Kasper J. , Freedman V., Spillman B. (2013). *Classification of persons by dementia status in the national health and aging trends study*. Johns Hopkins Bloomberg School of Public Health.

[gbaf147-B24] Keith C. (1995). Family caregiving systems: Models, resources, and values. Journal of Marriage and the Family, 57, 179. 10.2307/353826

[gbaf147-B25] Lai W. , Nemmers N., Tsuker S., Leggett A. N. (2025). Exploring caregiving network characteristics for older adults living with cognitive impairment across race and ethnicity. The Gerontologist, 65, gnaf110. 10.1093/geront/gnaf110 PMC12129109 40096543

[gbaf147-B26] Leggett A. N. , Strominger J., Robinson-Lane S. G., Maust D. T. (2022). Disparities in health care task participation and provider communication by family caregiver race. Journal of General Internal Medicine, 37, 1321–1324. 10.1007/s11606-021-06766-w 33830417 PMC8971267

[gbaf147-B27] Liang J. , Aranda M. P., Jang Y., Wilber K., Chi I., Wu S. (2022). The effect of support from secondary caregiver network on primary caregiver burden: do men and women, Blacks and Whites differ? The Journals of Gerontology, Series B: Psychological Sciences and Social Sciences, 77, 1947–1958. 10.1093/geronb/gbac067 35511820 PMC9535770

[gbaf147-B28] Lin Z. (2025). Racial-ethnic differences in care networks of older adults: empirical exploration of possible explanations. *The Journals of Gerontology, Series B: Psychological Sciences and Social Sciences*, 80, gbaf038. 10.1093/geronb/gbaf038 PMC12079420 39989205

[gbaf147-B29] Lin Z. , Liu H. (2024). Race/ethnicity, nativity, and gender disparities in unmet care needs among older adults in the United States. The Gerontologist, 64, gnad094. 10.1093/geront/gnad094 PMC10943507 37434547

[gbaf147-B30] Litwak E. (1985). Helping the elderly: The complementary roles of informal networks and formal systems. Guilford Press.

[gbaf147-B31] Litwak E. (1986). Helping the elderly: The complementary roles of informal networks and formal systems. Psychiatric Services, 37, 639–640. 10.1176/ps.37.6.639

[gbaf147-B32] Manalel J. A. , Antonucci T. C. (2022). Development of social convoys: Trajectories of convoy structure and composition from childhood through adulthood. Developmental Psychology, 58, 1806–1815. 10.1037/dev0001390 35653760 PMC9639451

[gbaf147-B33] Marcum C. S. , Ashida S., Koehly L. M. (2020). Primary caregivers in a network context. The Journals of Gerontology, Series B: Psychological Sciences and Social Sciences, 75, 125–136. 10.1093/geronb/gbx165 29304203 PMC7179806

[gbaf147-B34] Martinson M. , Berridge C. (2015). Successful aging and its discontents: A systematic review of the social gerontology literature. The Gerontologist, 55, 58–69. 10.1093/geront/gnu037 24814830 PMC4986586

[gbaf147-B35] Mendez-Luck C. A. , Anthony K. P. (2016). “Marianismo” and caregiving role beliefs among U.S.-born and immigrant Mexican women. The Journals of Gerontology, Series B: Psychological Sciences and Social Sciences, 71, 926–935. 10.1093/geronb/gbv083 26362602 PMC4982386

[gbaf147-B36] Miller K. , Yang Y., Reckrey J., Ornstein K., Wolff J. (2024). Trajectories of paid and family care to support adults with disabilities by rurality. Innovation in Aging, 8(Suppl_1), 518–519. 10.1093/geroni/igae098.1694

[gbaf147-B37] Mlinac M. E. , Feng M. C. (2016). Assessment of activities of daily living, self-care, and independence. Archives of Clinical Neuropsychology, 31, 506–516. 10.1093/arclin/acw049 27475282

[gbaf147-B38] Moon H. E. , Rote S. M., Sears J., Schepens Niemiec S. L. (2022). Racial differences in the dementia caregiving experience during the COVID-19 pandemic: Findings from the National Health and Aging Trends Study (NHATS). The Journals of Gerontology, Series B: Psychological Sciences and Social Sciences, 77, e203–e215. 10.1093/geronb/gbac098 35869747 PMC9384524

[gbaf147-B39] Nkimbeng M. J. , Parker L. J. (2021). Diverse, culturally rich approaches to family care in the United States. In Bridging the Family Care Gap (pp. 43–69). Elsevier. 10.1016/B978-0-12-813898-4.00002-6

[gbaf147-B40] Okoro C. A. , Hollis N. D., Cyrus A. C., Griffin-Blake S. (2018). Prevalence of disabilities and health care access by disability status and type among adults–United States, 2016. Morbidity and Mortality Weekly Report, 67, 882–887. 10.15585/mmwr.mm6732a3 30114005 PMC6095650

[gbaf147-B41] Parker L. J. , Fabius C. (2022). Who’s helping whom? examination of care arrangements for racially and ethnically diverse people living with dementia in the community. Journal of Applied Gerontology, 41, 2589–2593. 10.1177/07334648221120247 35960528 PMC10348595

[gbaf147-B42] Patterson S. E. , Margolis R. (2023). Family ties and older adult well-being: incorporating social networks and proximity. The Journals of Gerontology, Series B: Psychological Sciences and Social Sciences, 78, 2080–2089. 10.1093/geronb/gbad139 37738615 PMC10699742

[gbaf147-B43] Power M. C. , Bennett E. E., Turner R. W., Dowling N. M., Ciarleglio A., Glymour M. M., Gianattasio K. Z. (2021). Trends in relative incidence and prevalence of dementia across non-Hispanic Black and White individuals in the United States, 2000-2016. JAMA Neurology, 78, 275. 10.1001/jamaneurol.2020.4471 33252617 PMC7953306

[gbaf147-B44] Shang Y. , Patterson S. E. (2024). Confidants and caregivers: Network multiplexity and subjective well-being of older adults. The Journals of Gerontology, Series B: Psychological Sciences and Social Sciences, 79, gbae164. 10.1093/geronb/gbae164 PMC11528350 39330253

[gbaf147-B45] Shaw A. L. , Riffin C. A., Shalev A., Kaur H., Sterling M. R. (2021). Family caregiver perspectives on benefits and challenges of caring for older adults with paid caregivers. Journal of Applied Gerontology, 40, 1778–1785. 10.1177/0733464820959559 32975471 PMC7990746

[gbaf147-B46] Song M.-K. , Paul S., Happ M. B., Lea J., Pirkle J. L., Turberville-Trujillo L. (2023). Informal caregiving networks of older adults with dementia superimposed on multimorbidity: A social network analysis study. Innovation in Aging, 7. igad033. 10.1093/geroni/igad033 PMC10184695 37197444

[gbaf147-B47] Spillman B. C. , Allen E. H., Favreault M. (2021). *Informal caregiver supply and demographic changes: Review of the literature*. U.S Department of Health and Human Services.

[gbaf147-B48] Spillman B. C. , Freedman V. A., Kasper J. D., Wolff J. L. (2020). Change over time in caregiving networks for older adults with and without dementia. The Journals of Gerontology, Series B: Psychological Sciences and Social Sciences, 75, 1563–1572. 10.1093/geronb/gbz065 31102533 PMC7424285

[gbaf147-B49] Taylor R. J. , Chatters L. M., Skipper A. D., Ellis J. (2021). Older African American, Black Caribbean, and Non-Latino White fictive kin relationships. Annual Review of Gerontology & Geriatrics, 41, 1–31. PMC9005029 35418718

[gbaf147-B50] U.S. Census Bureau. (2023). *Projections for the United States: 2023 to 2100*. https://www.census.gov/data/datasets/2023/demo/popproj/2023-popproj.html

[gbaf147-B51] U.S. Census Bureau. (2025). *Poverty thresholds*. https://www.census.gov/data/tables/time-series/demo/income-poverty/historical-poverty-thresholds.html

[gbaf147-B52] Van Houtven C. H. , Konetzka R. T., Taggert E., Coe N. B. (2020). Informal and formal home care for older adults with disabilities increased, 2004–16. Health Affairs, 39, 1297–1301. 10.1377/hlthaff.2019.01800 32744936 PMC8729158

[gbaf147-B53] Verdery A. M. , Margolis R. (2017). Projections of White and Black older adults without living kin in the United States, 2015 to 2060. Proceedings of the National Academy of Sciences of the United States of America, 114, 11109–11114. 10.1073/pnas.1710341114 28973934 PMC5651770

[gbaf147-B54] Wolff J. L. , Cornman J. C., Freedman V. A. (2025). The number of family caregivers helping older US adults increased from 18 million To 24 million, 2011–22. Health Affairs, 44, 187–195. 10.1377/hlthaff.2024.00978 39899774 PMC11869104

[gbaf147-B55] York Cornwell E. , Goldman A. W. (2021). Local ties in the social networks of older adults. The Journals of Gerontology, Series B: Psychological Sciences and Social Sciences, 76, 790–800. 10.1093/geronb/gbaa033 32227105 PMC7955986

