# Supplementary material for: Evolution of shared care networks by race and ethnicity: findings from the National Health and Aging Trends Study
Source: J Gerontol B Psychol Sci Soc Sci. 2025 Aug 7;80(10):gbaf147. doi: 10.1093/geronb/gbaf147 (PMC12462773; doi:10.1093/geronb/gbaf147)
Supplement: gbaf147_Supplementary_Data [file gbaf147_supplementary_data.docx]

**Appendices**

Appendix A. Assembly of the Analytic Sample from the 2015 & 2022 National Health and Aging Trends Study


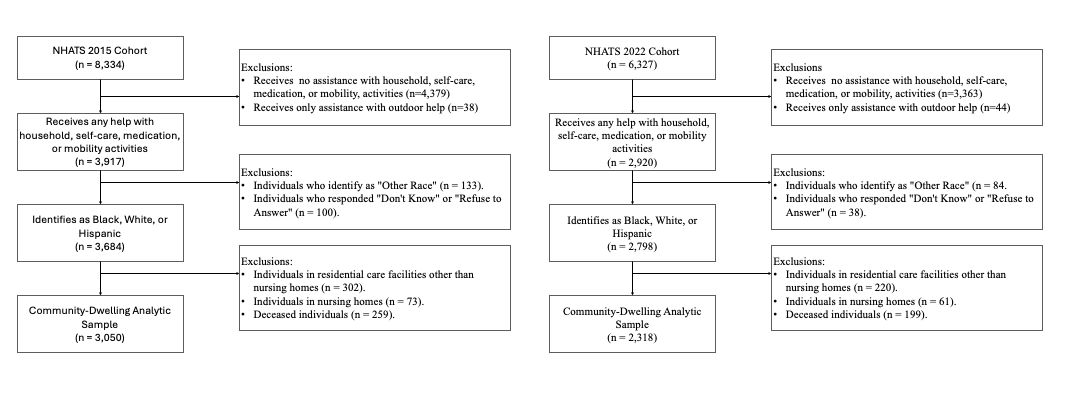


| Appendix B. Sociodemographic Characteristics of Care Helpers in 2015 and 2022 | | | | | | | | | | | | | | | | | |
| --- | --- | --- | --- | --- | --- | --- | --- | --- | --- | --- | --- | --- | --- | --- | --- | --- | --- |
|  | 2015 | | | | | | | | 2022 | | | | | | | | |
|  | White | Black | | Hispanic | | p-value | | White | | | Black | | Hispanic | | p-value | |  |
| Weighted estimates (*thousands*) | 22227.31 | 3287.18 | | 3100.79 | |  | | 23970.48 | | | 4160.61 | | 3905.87 | |  | |  |
| Unweighted (n) | 4,349 | 1,890 | | 574 | |  | | 2,830 | | | 1,442 | | 798 | |  | |  |
| *Helper Relationship %, (n)* |  |  | |  | |  | |  | | |  | |  | |  | |  |
| Spouse / Partner | 31.6  (1134) | 15.9  (232) | | 24.0  (103) | | 0.00 | | 32.8  (763) | | | 14.7  (169) | | 20.3  (143) | | 0.00 | |  |
| Child | 36.1  (1776) | 44.0  (867) | | 47.9  (300) | |  |  | 30.6  (1098) | | | 38.1  (626) | | 47.8  (410) | |  |  |  |
| Other Family | 11.0  (494) | 20.9  (424) | | 12.7  (83) | |  |  | 13.5  (366) | | | 26.0  (355) | | 19.3  (142) | |  |  |  |
| Non-Relative | 21.2  (944) | 19.1  (365) | | 15.4  (88) | |  |  | 23.1  (602) | | | 20.99  (291) | | 12.6  (102) | |  |  |  |
| *Gender %, (n)* |  |  | |  | |  |  |  | | |  | |  | |  | |  |
| Female | 56.6  (2455) | 59.5  (1140) | | 59.1  (350) | | 0.01 | | 56.3  (1651) | | | 61.5  (868) | | 58.9  (462) | | 0.43 | |  |
| *Proximity %, (n)* |  |  | |  | |  | |  | | |  | |  | |  | |  |
| Lives in Household | 45.1  (1748) | 39.2  (685) | | 49.9  (269) | | 0.00 | | 46.7  (1175) | | | 34.9  (492) | | 51.8  (398) | | 0.15 | |  |
| *Helper is Paid %, (n)* |  |  | |  | |  | |  | | |  | |  | |  | |  |
| Overall | 12.4  (582) | 11.6  (231) | | 13.7  (84) | | 0.26 | | 12.5  (368) | | | 14.0  (219) | | 12.9  (112) | | 0.14 | |  |
| Child | 8.0  (53) | 10.8  (27) | | 20.2  (19) | | 0.00 | | 8.0  (31) | | | 20.9  (37) | | 37.7  (38) | | 0.00 | |  |
| Other Family | 5.1  (35) | 10.3  (24) | | 5.6  (6) | | 0.22 | | 3.4  (17) | | | 17.4  (41) | | 8.6  (10) | | 0.00 | |  |
| Non-Relative | 86.9  (494) | 79.0  (180) | | 74.2  (59) | | 0.09 | | 88.4  (319) | | | 61.7  (141) | | 53.7  (64) | | 0.02 | |  |
|  |  | |  | |  | |  | | |  | |  | |  | |  |  |

**Data are survey-weighted adjusted. Cases with missing data for Helper Relationship are excluded (n=1) for White sampled persons (2015 and 2022); (n=1) for Black sampled persons (2022)*

| Appendix C. Number of Helpers for Each Care Recipient By Task Domain | | | | | | | | | | |
| --- | --- | --- | --- | --- | --- | --- | --- | --- | --- | --- |
|  | 2015 | | | | | 2022 | | | | |
|  | White | Black | Hispanic | p-value | White | | Black | Hispanic | p-value |  |
| Unweighted (n) | 2039 | 755 | 256 |  | 1,377 | | 580 | 361 |  |  |
| Household-Related Assistance (%, n) | | | | | | | | | |  |
| 0 | 7.0  (149) | 7.7  (56) | 5.5  (13) | 0.00 | 8.2  (113) | | 5.6  (41) | 5.8  (22) | 0.00 |  |
| 1 | 69.2  (1341) | 60.4  (429) | 71.2  (171) |  | 70.4  (915) | | 61.1  (322) | 60.9  (218) |  |  |
| 2+ | 23.8  (549) | 31.9  (270) | 23.2  (72) |  | 21.4  (349) | | 33.3  (217) | 33.4  (121) |  |  |
| Self-Care Related Assistance (%, n) | | | | | | | | | |  |
| 0 | 71.0  (1409) | 63.7  (456) | 61.9  (148) | 0.00 | 72.1  (957) | | 66.3  (358) | 59.8  (222) | 0.00 |  |
| 1 | 22.8  (471) | 26.7  (208) | 30.1  (78) |  | 23.4  (332) | | 24.0  (153) | 28.7  (94) |  |  |
| 2+ | 6.2  (159) | 9.6  (91) | 8.0  (30) |  | 4.5  (88) | | 9.7  (69) | 11.5  (45) |  |  |
| Mobility-Related Assistance | | | | | | | | | |  |
| 0 | 76.7  (1507) | 68.5  (489) | 66.1  (156) | 0.00 | 74.0  (994) | | 62.7  (338) | 52.9  (187) | 0.00 |  |
| 1 | 13.5  (288) | 15.7  (128) | 21.2  (56) |  | 17.4  (234) | | 19.8  (116) | 28.2  (100) |  |  |
| 2+ | 9.7  (244) | 15.9  (138) | 12.8  (44) |  | 8.6  (149) | | 17.7  (126) | 18.8  (74) |  |  |
| Medical-Related Assistance (%, n) | | |  |  |  | |  |  |  |  |
| 0 | 15.5  (290) | 24.2  (159) | 13.0  (31) | 0.00 | 23.5  (269) | | 28.1  (134) | 21.8  (69) |  |  |
| 1 | 73.6 (1476) | 61.4  (463) | 72.9  (176) |  | 68.0  (952) | | 59.5  (259) | 64.8  (240) | 0.02 |  |
| 2+ | 10.9  (273) | 14.4  (133) | 14.1  (49) |  | 8.6  (156) | | 12.4  (87) | 13.4  (52) |  |  |
